# Intra-operative computer navigation guided cervical pedicle screw insertion in thirty-three complex cervical spine deformities

**DOI:** 10.4103/0974-8237.65480

**Published:** 2010

**Authors:** S. Rajasekaran, P. Rishi Mugesh Kanna, T. Ajoy Prasad Shetty

**Affiliations:** Department of Orthopaedics, Traumatology and Spine Surgery, Ganga Hospital, 313, Mettupalayam Road, Coimbatore-641 043, India

**Keywords:** Cervical spine, navigation, pediatric, pedicle screws

## Abstract

**Background:**

Cervical pedicle screw fixation is challenging due to the small osseous morphometrics and the close proximity of neurovascular elements. Computer navigation has been reported to improve the accuracy of pedicle screw placement. There are very few studies assessing its efficacy in the presence of deformity. Also cervical pedicle screw insertion in children has not been described before. We evaluated the safety and accuracy of Iso-C 3D-navigated pedicle screws in the deformed cervical spine.

**Materials and Methods:**

Thirty-three patients including 15 children formed the study group. One hundred and forty-five cervical pedicle screws were inserted using Iso-C 3D-based computer navigation in patients undergoing cervical spine stabilization for craniovertebral junction anomalies, cervico-thoracic deformities and cervical instabilities due to trauma, post-surgery and degenerative disorders. The accuracy and containment of screw placement was assessed from postoperative computerized tomography scans.

**Results:**

One hundred and thirty (89.7%) screws were well contained inside the pedicles. Nine (6.1%) Type A and six (4.2%) Type B pedicle breaches were observed. In 136 levels, the screws were inserted in the classical description of pedicle screw application and in nine deformed vertebra, the screws were inserted in a non-classical fashion, taking purchase of the best bone stock. None of them had a critical breach. No patient had any neurovascular complications.

**Conclusion:**

Iso-C navigation improves the safety and accuracy of pedicle screw insertion and is not only successful in achieving secure pedicle fixation but also in identifying the best available bone stock for three-column bone fixation in altered anatomy. The advantages conferred by cervical pedicle screws can be extended to the pediatric population also.

## INTRODUCTION

The management of cervical spine deformities has certain inherent problems due to the smaller size of the osseous elements, inconsistent anatomical landmarks in complex congenital anomalies and the close proximity of vital neurovascular structures.[1] Further, in the pediatric population, the fusion potential is decreased due to the poor healing potential of the immature bone, increased segmental mobility and poor patient compliance.[2] Rigid spinal stabilization in these patients would help in deformity correction and decrease the pseudoarthrosis rate. However, traditional stabilization techniques like loops, spinous process/ facet wiring techniques and lateral mass screws have a poor healing potential and need to be supported with external orthosis.[3] But halo vests themselves have a high morbidity rate with frequent failures and revision surgeries are fraught with further complications.[4]

Pedicle screw instrumentation is the preferred method of posterior stabilization in the thoracic and lumbar spine.[5] It has the advantages of three-column fixation, better deformity correction, lower incidence of implant failures and pseudoarthrosis.[5] But unlike the thoraco-lumbar spine, cervical pedicle screw systems have not gained much popularity. Abumi *et al*., reported the successful use of cervical pedicle screws in various conditions like traumatic disorders, cervical kyphosis and spondyloarthropathies.[6,7] But other studies have shown a high incidence of neural and vascular injury during cervical pedicle screw insertion.[8] Further, in children, the anatomical size constraints, unreliable anatomical landmarks, the variable location of the transverse foramen and other unpredictable osseous morphology in congenital cervical anomalies place the vital neurovascular structures at considerable risk in the bargain for rigid skeletal fixation.

Computer navigation has been found to improve the accuracy of pedicle screw fixation with significantly reduced misplacement rates.[9,10‐16] Navigation has been shown to increase the success rate of pedicle screw insertion in adults by up to 97%[12]. To the best of our knowledge, insertion of cervical pedicle screws in the pediatric population has not been studied before. Also, there are very few studies[12,13,17] reporting on the efficacy of computer navigation in cervical spine deformity correction. Hence we prospectively analyzed the accuracy of cervical pedicle screw placement using Iso-C 3D-based navigation in both children and adults.

## MATERIALS AND METHODS

Thirty-three patients with deformities of the cervical spine of varying etiology underwent cervical spine stabilization by pedicle screw fixation using intra-operative computer navigation. The etiology of the deformities is seen in Table 1.

**Table 1:** Etiology of cervical spine deformities

Etiology of deformities	No. of patients (n=33)
Arnold Chiari malformation	2
Basilar invagination	3
Os odontoideum	2
Atlanto-axial subluxation	4
Traumatic C1-2 instability	2
C1-2 rotary subluxation	2
Degenerative C1-2 arthritis	2
Rheumatoid C1-2 instability	1
Post-tubercular deformity/ instability	2
Degenerative sub-axial instability with stenosis	3
Traumatic sub-axial fractures	3
Cervical thoracic deformity	4
Cervical tumor excision	3

**Figure 1:**
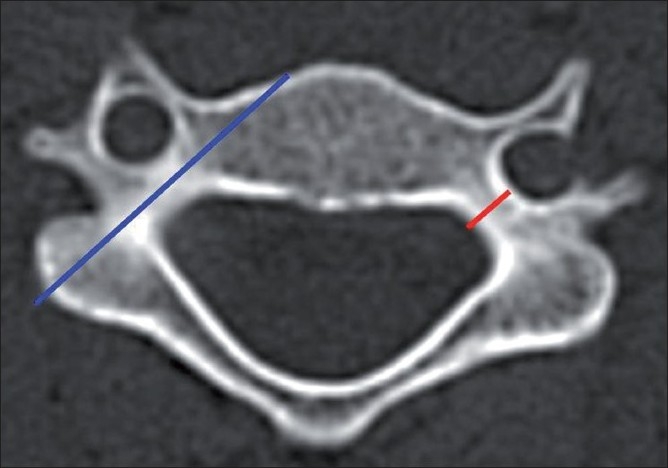
Technique of measuring the pedicle width and the axis length in the axial CT image before planning screw insertion. The pedicle width (red line) is measured at its narrowest part between the two cortices. The pedicle axis length (blue line) is measured along its axis from the lateral mass till the anterior vertebral body

Radiologic evaluation included cervical radiographs in two diagonal planes, fine 2-mm computerized tomography (CT) cuts with multi-planar reconstruction and magnetic resonance imaging (MRI) with a 1.5 Tesla scanner to assess the possibility of application of cervical pedicle screws at each level. CT scan images were analyzed for anomalies of the vertebra, dimensions of the deformity and special attention was directed to the pedicle width at its narrowest part and the length of the pedicle axis at each level [Figure 1]. The pedicle width was measured to assess the feasibility of screw insertion. In patients with atypical vertebral morphology, an adequate (>4.5 mm width) segment of bone contiguous in the three spinal columns was selected for screw insertion. The pedicle axis length was assessed to determine the length of the screw possible at each level. The mean age of the patient was 24.2 ± 6.2 years (range 3-52 years). Standard 3.5 mm titanium pedicle polyaxial screws were inserted in all patients with Iso-C 3D computer navigation (Brainlab, *Vector Vision*). All screws were inserted through the same incision as against separate stab incisions. The technique of navigation-guided screw insertion is given below. The screws were interconnected with titanium solid rods. The posterior elements were prepared for grafting with a burr and posterior iliac crest autograft was placed. Patients were mobilized with a soft cervical collar for comfort which was worn for six to eight weeks.

### Surgical technique

**Figure 2:**
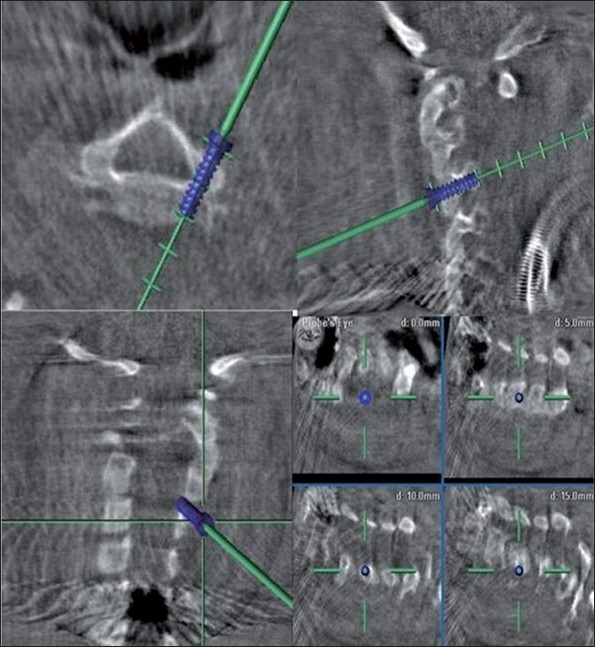
Intra-operative navigation images showing the ideal screw trajectory, planned screw width and length in multiple planes (axial, sagittal, coronal and oblique sections)

The patients were operated in the prone position on a carbon top radiolucent table (Hobo, China). The spine was exposed posteriorly through a standard midline approach and the minimally invasive reference array (MIRA) was attached to the spinous process of a vertebra just caudal to the last instrumented vertebra. The Siremobil Iso C^3D^ (Seimens, Germany) image intensifier obtained fluoroscopic images of the relevant segments of the spine by moving through an arc around the spine. With a single registration, it was usually possible to include the entire cervical spine in children and five to six levels in adults. The acquired data was then transferred to the computer navigation platform - Vector Vision (Spine version 2.0 Brain Lab, Germany) which reconstructed the data to provide real-time intra-operative multi-planar images of the vertebra. The tool navigator provided 3D virtual images which helped in identifying the screw entry point, trajectory and the pedicle dimensions [Figure 2]. The dorsal cortex at the identified entry site was burred and the pedicle was gently negotiated using a sharp pedicle finder. The direction and depth of the pedicle was confirmed frequently using the tool navigator. Polyaxial 3.5 mm titanium screws were used in all patients. Time required for data acquisition was divided by the total number of screws inserted in each patient. The average time needed for setup per screw was added to the time taken for actual screw insertion, which then gave the insertion time for that particular screw.

**Figure 3:**
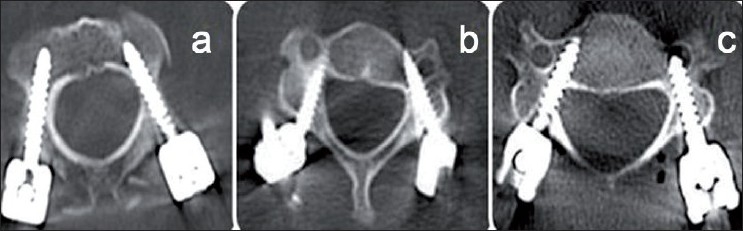
The three types of pedicle perforation. (a): correct screw placement without pedicle perforation or with pedicle perforation <1.0 mm (depth of the thread); (b): pedicle perforation >1.0 mm or screw tip encroaching the vertebral foramen but without the need for any screw revision and (c): pedicle perforation >1.0 mm with the need for screw revision because of irritation or injury of roots or the spinal cord, or because of reduced biomechanical stability

The postoperative CT scans were analyzed for breach of the pedicle wall by the screw, either medially, laterally, inferiorly, or superiorly. The containment of screws was assessed by multi-planar 1.5 mm reconstructions in each screw axis. The screw position was divided into three groups: Group A, correct screw placement without pedicle perforation or with pedicle perforation <1.0 mm (depth of the thread); Group B, pedicle perforation >1.0 mm or screw tip encroaching the vertebral foramen but without the need for any screw revision and Group C, pedicle perforation >1.0 mm with the need for screw revision because of irritation or injury of roots or the spinal cord, or because of reduced biomechanical stability [Figure 3a‐c]. CT scans of the cervical spine were also performed at either six or 12 months after surgery. Presence of a bridging posterior fusion mass was considered as an indicator of fusion.

## RESULTS

**Figure 4:**
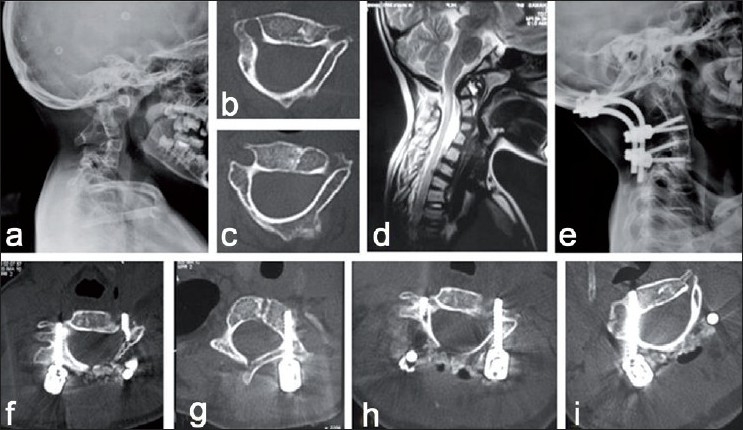
Cervical pedicle screw-instrumented occipito-cervical fusion in an eight-year-old child. (a): Lateral radiograph of the cervical spine showing cervical segmentation anomaly with atlanto-axial instability. (b and c): Axial CT images showing atypical attenuated dysmorphic pedicles at C3 and C4 levels (d): Sagittal MRI of the cervical spine showing atlanto-axial instability with cord compression at the craniovertebral junction. (e): Lateral X-ray of the cervical spine shows reduction of atlanto-axial instability with subaxial cervical pedicle screws and occipito-cervical fusion. (f-i): Axial CT images showing well-contained cervical pedicle screws without any pedicle breach at C3, C4 levels

**Figure 5:**
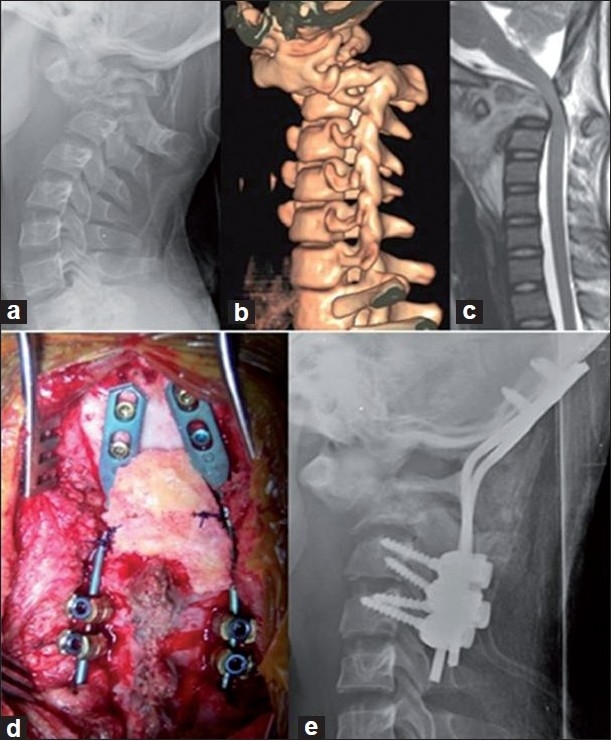
Atlanto-axial tuberculosis in a 12-year-old girl. (a): Lateral radiograph showing destroyed C2 with instability. (b): 3D CT scan showing the dislocation. (c): Sagittal T2 MRI shows cord compression. (d, e): Intra-operative picture and postoperative radiograph showing the final construct of occipito-cervical fixation with cervical pedicle screws

One hundred and forty-five cervical pedicle screws were inserted in all, out of which 98 (67.5%) were in adult cervical vertebra and 47 (32.4%) were in children. The level-wise distribution of screws is as follows: C1- 24, C2- 45, C3- 27, C4- 12, C5- 12, C6- 7 and C7- 18. In none of the cases, was there any change in the operative plan to skip a level, change the screws or an alternate method of fixation used. There was a good agreement between the preoperative CT images and the intraoperative Iso-C-based images. In several deformed vertebrae due to the presence of atypical pedicles with inconsistent landmarks (n=9), the screws were inserted in a non-classical fashion to achieve the best bone purchase in the three columns of the spine [Figure 4]. But at all other cervical pedicles (n=136), the screws were inserted in the standard manner based on described entry points and trajectory, with computer navigation assistance [Figure 5].

One hundred and thirty screws (89.7%) were perfectly contained inside the pedicles. There were 15 pedicle breaches (10.3%), which included nine Type A and six Type B perforation. The main pedicle breach was on the medial wall (7/145) followed by lateral wall (5/145), superior pedicle (2/145) and anterolateral body (1/145). The grade of pedicle breach is shown in Table 2. Neither excessive intra-operative bleeding nor any signs of vertebrobasilar insufficiency were observed postoperatively in patients who had a lateral pedicle breach. No nerve root or spinal cord injury was encountered. Since the screws were biomechanically stable with good purchase in the lateral mass and the vertebral body, they were not revised. No specific reasons could be ascertained for these perforations as the pedicle widths were not small or restrictive as compared to the other levels. A technical error in navigation registration or screw insertion is a possibility. Out of 47 pedicle screws inserted in children, six had a pedicle breach and all of them were Type B perforations (12.8%). Comparatively, in adults, out of the 98 screws inserted, only nine had a pedicle breach (9.2%) and only two were Type B. Average pedicle screw insertion time in cervical spine was 2.4 +/_ 0.3 min. In our series, there were no incidences of Type C pedicle breaches and none of the screws were revised. The mean follow-up of the patients was 18.3 ± 12.1 months (range: 6-40 months).

Preoperatively all of them had normal neurological status except for two patients. The first patient had a basilar invagination and presented late with worsening neurological status (American Spinal Injury Association Scale, ASIA C). He underwent a posterior decompression, distraction and instrumented occipto-cervical fusion. Postoperatively his respiratory effort was poor and had a protracted recovery in the intensive care support. He improved gradually and regained ambulatory status at three months after surgery. Another child presented with worsening neurological symptoms due to congenital kyphoscoliosis (ASIA C). She underwent first-stage posterior pedicle screw stabilization and was planned for decompression in the second stage. However, she worsened neurologically two days after the procedure due to suspected hypoxia following Acute Respiratory Distress Syndrome No evidence of spinal cord compression could be identified and she did not regain her walking strength. The other patients had normal neurological status postoperatively.

**Table 2:** Data on the incidence and grade of pedicle breach

Type of breach	Medial pedicle	Lateral pedicle	Superior pedicle	Inferior pedicle	Anterolateral body
Type A	2	4	-	-	1
Type B	5	1	2	-	-
Type C	-	-	-	-	-

## DISCUSSION

Pedicle screw stabilization has been proved to be biomechanically superior to other methods of spinal fixation through virtue of its three-column purchase.[18] Though commonly used in the lumbar and thoracic spine, its use in the cervical spine is limited for fear of inadvertent neuro-vascular injury.[19] Pedicle screw instrumentation of the cervical spine is a challenging procedure due to various factors like the narrow diameter of the pedicles, the variations in pedicle angulations and offsets as well as anatomical variations in the course and size of the vertebral artery.[20‐23] The presence of deformity and altered anatomy due to congenital anomalies further adds to the difficulty. When the insertion of pedicle screws in the adult cervical spine is considered difficult, its application in pediatric patients considering their small bone morphometrics is even more demanding. Hence its advantages need to be weighed against the potential risks to neurovascular structures. However to the best of our knowledge, there is no published literature regarding the use of pediatric cervical pedicle screws. This is the first time in literature that we are reporting the possibility, safety and technique of cervical pedicle screw application in the pediatric population.

Various methods have been advised for cervical pedicle screw insertion like the conventional technique using anatomical landmarks, foramino-laminotomy technique or with the assistance of computer navigation. Studies on the conventional technique of pedicle screw placement have reported pedicle violation rates ranging from 6.7–12% and an incidence of neurological injury ranging from 0–1.7%.[19,24‐26] Though the foramino-laminotomy technique has been advised to increase the accuracy of screw placement, pedicle violation rates even up to 55% have been reported.[26] The open lamina technique requires surgical exposure of the epidural space and hence can potentially destabilize the spine, reduce the posterior bone bed available for fusion, result in increased operative time, risk of dural tear, and blood loss.[27] Computer navigation is advised to improve the accuracy of pedicle screw insertion and studies comparing pedicle screw placement using conventional and computer navigation techniques have demonstrated the superior accuracy of this technology.[11,12,16] However, there are very few studies documenting the efficacy of computer navigation in pedicle screw placement in deformed cervical spine, especially of the pediatric sub-axial cervical spine.[11,12,16]

In the present study, there were 15 pedicle breaches (10.3%), but none had injuries to the vertebral artery, spinal cord or nerve root. Even with documented lateral wall perforation, no complications involving the vertebral artery were clinically apparent, both intra and postoperatively. Anatomical studies have shown that minimal violations of the foramen transversarium may not be as risky as the vertebral artery does not occupy the whole of the foramen transversarium.[25,26] Richter *et al*., reported 8.6% of pedicle screw perforation with conventional technique and 3% with CT-based navigation-assisted pedicle screw fixation.[28] Abumi *et al*., in 1997 reported only a 6.7% misplacement rate (out of 669 cervical pedicle screws) when using a conventional screw insertion technique.[24] Further, in a comparative study between the conventional and computer-navigated technique for cervical pedicle screw insertion, the rate of pedicle wall perforation was observed to be significantly lower in the computer-assisted group (1.2%). Similar to the present study, neuro-vascular complications were not demonstrated in the computer-assisted group.[12] This further underlines the fact that computer navigation improves the accuracy of screw placement. The higher rate of pedicle breaches seen in the present series may be due to the presence of congenital anomalies and inclusion of pediatric cervical deformities in the study group.

The potential risk of canal stenosis due to pedicle screw insertion in children seems largely theoretical.[29] Several studies analyzing the growth of thoracic and lumbar vertebra have shown that the vertebral canal dimensions reach more than 80% of their adult levels even by three to four years of age.[29,30] Further increase in the vertebral size is contributed mainly by the growth of the vertebral body alone. In a pediatric cadaver skeleton study, Vara *et al*., measured the mean cervical pedicle and canal measurements at each cervical level and concluded that the antero-posterior spinal canal diameter remains constant with increasing age whereas the medio-lateral canal diameter reaches 80% of adult dimensions by three to four years of age.[9] So the linear pedicle growth is very little and the spinal canal matures quite early in childhood. Hence injury and interference with the growth of the neural synchondrosis does not cause spinal stenosis or retarded vertebral growth and this should allay any fear of growth disturbance due to cervical pedicle screws.

In upper cervical instabilities, the anatomy can be quite distorted in many instances, where pedicle screws by conventional techniques would be difficult and not possible. For good access to the C1 lateral mass, the C2 ganglion also needs to be sacrificed.[31] In a multicentric study on C1-C2 fusion in 102 adult patients, Aryan *et al*., were not able to identify the C2 pedicle in 23 patients (22%). The vertebral artery also had a variable course in more than 25% of these patients, forcing them to use alternative methods of fixation.[31] On the contrary, with the use of computer navigation, the entry point is determined by the navigation probe. This obviates the need to widely expose the anatomical landmarks and thus helps to avoid sacrificing the C2 ganglion. Similarly, even when the gross vertebral anatomy was altered in two patients, navigation was useful in identifying the rudimentary pedicles so that screws could be put avoiding the spinal canal or the foramen transversarium.

Computer navigation can have its pitfalls too. In case of osteoporosis, the image quality can be poor reducing the surgeon's ability to identify the correct entry point and trajectory. Moreover, navigation is a computer-based software and the system can collapse at any time. Like all image-guidance methods, the Iso-C cannot substitute anatomic knowledge of the spine, especially in the three-dimensionally complex cervical spine. Hence it should not be used as a substitute for surgical judgment and experience, but rather as a tool to enhance the surgeon's safety while planning cervical screws.

## CONCLUSION

Intra-operative computer-based navigation improves the safety and accuracy of pedicle screw insertion and is a vital tool when planning surgeries for deformities of the cervical spine. It can very well be used in the pediatric cervical spine, where it can be advantageous in reducing the extent of instrumentation, improving the fusion rates with minimal morbidity.
